# Thermal Imaging Is a Noninvasive Alternative to PET/CT for Measurement of Brown Adipose Tissue Activity in Humans

**DOI:** 10.2967/jnumed.117.190546

**Published:** 2018-03

**Authors:** James Law, David E. Morris, Chioma Izzi-Engbeaya, Victoria Salem, Christopher Coello, Lindsay Robinson, Maduka Jayasinghe, Rebecca Scott, Roger Gunn, Eugenii Rabiner, Tricia Tan, Waljit S. Dhillo, Stephen Bloom, Helen Budge, Michael E. Symonds

**Affiliations:** 1 Early Life Research Unit, Division of Child Health, Obstetrics, and Gynaecology, School of Medicine, University of Nottingham, Nottingham, United Kingdom; 2 Bioengineering Research Group, Faculty of Engineering, University of Nottingham, Nottingham, United Kingdom; 3 Division of Diabetes, Endocrinology, and Metabolism, Imperial College, London, United Kingdom; 4 Imanova Centre for Imaging Sciences, Imperial College, London, United Kingdom; 5 Centre for Neuroimaging Sciences, King’s College, London, United Kingdom; and; 6 Nottingham Digestive Disease Centre and Biomedical Research Centre, School of Medicine, University of Nottingham, Nottingham, United Kingdom

**Keywords:** brown adipose tissue, thermal imaging, infrared thermography, PET/CT

## Abstract

Obesity and its metabolic consequences are a major cause of morbidity and mortality. Brown adipose tissue (BAT) utilizes glucose and free fatty acids to produce heat, thereby increasing energy expenditure. Effective evaluation of human BAT stimulators is constrained by the current standard method of assessing BAT—PET/CT—as it requires exposure to high doses of ionizing radiation. Infrared thermography (IRT) is a potential noninvasive, safe alternative, although direct corroboration with PET/CT has not been established. **Methods:** IRT and ^18^F-FDG PET/CT data from 8 healthy men subjected to water-jacket cooling were directly compared. Thermal images were geometrically transformed to overlay PET/CT-derived maximum intensity projection (MIP) images from each subject, and the areas with the most intense temperature and glucose uptake within the supraclavicular regions were compared. Relationships between supraclavicular temperatures (T_SCR_) from IRT and the metabolic rate of glucose uptake (MR(gluc)) from PET/CT were determined. **Results:** Glucose uptake on MR(gluc)_MIP_ was found to correlate positively with a change in T_SCR_ relative to a reference region (*r*^2^ = 0.721; *P* = 0.008). Spatial overlap between areas of maximal MR(gluc)_MIP_ and maximal T_SCR_ was 29.5% ± 5.1%. Prolonged cooling, for 60 min, was associated with a further T_SCR_ rise, compared with cooling for 10 min. **Conclusion:** The supraclavicular hotspot identified on IRT closely corresponded to the area of maximal uptake on PET/CT-derived MR(gluc)_MIP_ images. Greater increases in relative T_SCR_ were associated with raised glucose uptake. IRT should now be considered a suitable method for measuring BAT activation, especially in populations for whom PET/CT is not feasible, practical, or repeatable.

Obesity is a leading global health concern, with no effective drug treatments currently available. An adverse metabolic profile, including insulin resistance and raised blood lipids, is a common pathway to many of the consequences of obesity. Brown adipose tissue (BAT) is a major effector of adaptive thermogenesis and an attractive antiobesity drug target (1). It is a highly metabolically active organ that utilizes glucose and free fatty acids and is able to release chemical energy efficiently as heat by uncoupling mitochondrial respiration from adenosine triphosphate production (2). Increased BAT activity results in raised energy expenditure, improved glycemic control, and an improved blood lipid profile (3).

In recent years, after the rediscovery of BAT in adult humans (4–8), it has been the focus of intense research. However, replication of the promising results from animal studies has been slow (9). This is due, in part, to difficulties in measuring BAT activity directly in humans. Because of the variable anatomic position of BAT, close to major vessels, safe and routine biopsy is difficult, causing imaging to become the preferred method of BAT quantification. The standard method of BAT imaging remains PET/CT, which exposes participants to a significant radiation dose and is therefore not suitable for studies with repeated measures, large numbers of healthy volunteers, or children. Measurements using PET/CT are also limited to fasting subjects (10).

An alternative imaging technique, infrared thermography (IRT), makes use of the heat-emitting properties of BAT and the relatively superficial position of the supraclavicular depot, one of the largest BAT depots in adults (7). Using IRT, several research groups have shown a specific rise in supraclavicular temperatures (T_SCR_) after introduction of a cool stimulus (11–14). IRT has the advantage of being able to measure real-time activation and can be used to gather repeated measures in large numbers of healthy subjects irrespective of age and nutritional status (11).

To date, IRT has not been validated against the current gold standard, ^18^F-FDG PET/CT. Although the results from multiple previous IRT studies are consistent with the measurement of BAT, the lack of data directly comparing results between PET/CT and IRT in the same subjects has remained a limitation of the technique. Using novel IRT analysis methods, as well as PET/CT analysis techniques additional to those used by Salem et al. (13), we explored the anatomic and functional relationship between alternative measures of BAT in individual participants. For the first time, to our knowledge, we show here that the area identified as overlying BAT using IRT closely corresponds to the area of maximal glucose uptake on PET/CT and that the higher the glucose uptake on PET/CT the better the agreement.

## MATERIALS AND METHODS

### Subjects

To determine the correlation between BAT activity measured by PET/CT and IRT, 8 healthy men known to be BAT-positive on PET/CT (13) (mean age, 23.5 y; range, 18–35 y; mean body mass index, 22.0 kg/m^2^; range 19.3–23.0 kg/m^2^) underwent PET/CT and IRT sessions as part of a study approved by the London Central Ethics and Research Committee (13/LO/0925), registered with ClinicalTrials.gov (NCT01935791), and performed in accordance with the Declaration of Helsinki. Written informed consent was obtained from subjects before enrollment in the study. Participants who were BAT-negative on the initial PET/CT scan (13) were not included in the analysis because of the small size of the group (*n* = 3).

### Study Visits

As previously described (13), participants attended an initial IRT session and an initial PET/CT session. For both visits, the volunteers wore a pair of light cotton trousers with a cooling vest surrounding the torso, away from the supraclavicular region. After acclimatization, cold water at 8°C (13) was pumped through the cooling vest to stimulate BAT.

**FIGURE 1.**
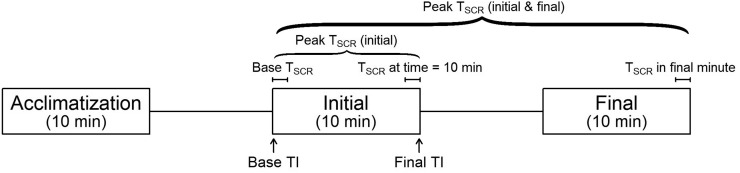
IRT imaging protocol annotated with IRT outcomes. Acclimatization = imaging period during acclimatization; initial = imaging period during first 10 min of stimulation; final = imaging period after stimulation for ∼1 h.

In summary, during the thermal imaging session, images were captured during acclimatization; over the first 10 min of cooling (initial period), which represents the period of maximal BAT activation from the resting state (12); and after prolonged stimulation (final period) (Fig. 1). Images were acquired using a FLIR T440bx infrared camera (FLIR Systems). During the PET/CT visit, 180 MBq of ^18^F-FDG were injected after water-vest cooling, and a 60-min dynamic emission scan was obtained with an axial field of view from mandible to mid thorax (13).

All volunteers attended a second thermal imaging session without cold stimulation (IRT vehicle session), and 4 volunteers who were BAT-positive on the first PET/CT scan underwent a further PET/CT imaging session, without cold stimulation (PET/CT vehicle session). Thermal and PET/CT images were acquired as before.

### Analysis of IRT

Thermal images (TIs) acquired by IRT at 5-s intervals were analyzed from each period of imaging for both IRT sessions. We developed a semiautomated method for analysis of TIs to allow the efficient analysis of large numbers of images in a systematic and reproducible manner by first converting the TIs to the nonproprietary Portable Network Graphic format, then identifying the supraclavicular regions of interest (ROIs), and finally processing the data within the ROIs.

The TIs were converted using our custom-built Thermal-Imaging Technical Conversion Hub, a Raspberry Pi–based device that extracts the raw data from the TI and saves it in an openly accessible format from which temperature data can be calculated (15,16). Our method for converting the raw radiometric data to temperature data produces results identical to those obtained by FLIR’s proprietary software (data not shown).

**FIGURE 2.**
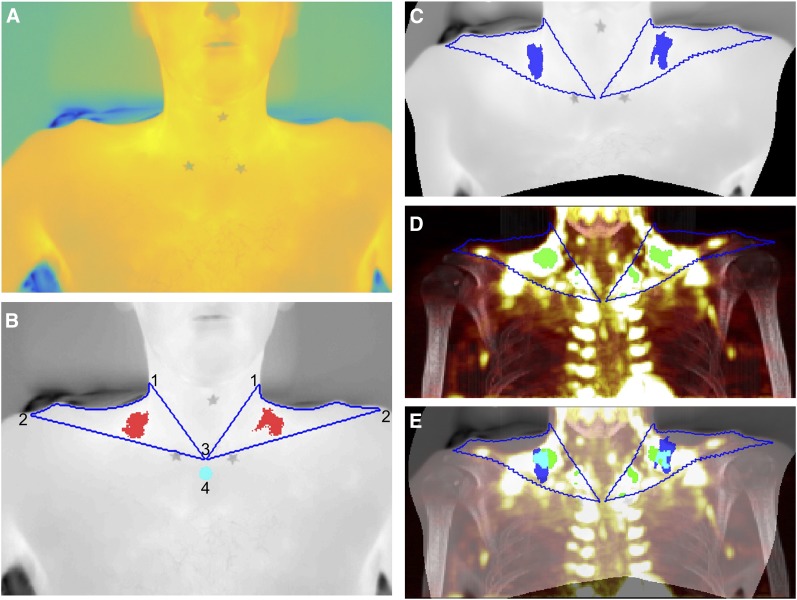
Mapping of TI to PET/CT. (A) Apices of ROIs identified on original TI. (B) Contour of ROI (blue) and hottest 10% of pixels (red) identified, with contour of neck precisely defined using automated process: left and right superolateral apices (1), acromioclavicular apices (2), sternal apex (3), and reference area (4). (C) TI after warping by lwm transformation calculated after identification of control-point pairs. (D) Warped contour superimposed on MIP with most intense 10% of pixels highlighted (green). (E) Final composite image with warped TI superimposed on MIP image and hottest pixels from TI (blue), most intense pixels from MIP (green), and overlap (cyan) demonstrating close anatomic proximity.

Left and right supraclavicular ROIs were defined as previously described (12). In contrast to previous methods (12), this simply required each image (Fig. 2A) to be labeled with 5 key points, corresponding to the apices of the ROIs (Fig. 2B). The medial and inferior borders of the ROI were defined as straight lines between the appropriate apices. The contour of the neck was defined programmatically by identifying the temperature gradient between the volunteer and the background. This approach represents a significant improvement on previously published methods, in which the lateral border of the ROI is approximated by a simple straight line (17) or is painstakingly identified between manually plotted points (12).

The hottest 10% of points within each ROI were identified, and the medians of these points were calculated (equivalent to the 95th percentile) (11,12,18). Corresponding graphical output allowed clear visualization of the identified hotspot (Fig. 2B). A reference region consisting of a circle 10 pixels in diameter immediately below the central apex of the ROIs was analyzed for comparison. A moving average (period 5) was applied to the resulting time series to reduce the effect of natural variation in measurements. The main outcome measures for IRT were base T_SCR_ (mean of the first minute of stimulation), peak T_SCR_ (maximal T_SCR_ within a given period), and ΔT_SCR_ (peak T_SCR_ – base T_SCR_), all calculated for the right ROI (11) over the first 10 min of cooling relative to the reference region (11,18). Secondary outcomes included analysis of absolute T_SCR_ values as well as comparison of left and right ROIs.

Videos of change in skin temperature over time, relative to the baseline image, were compiled. Pixels were averaged over 3 sequential images, and sequential frames were registered to the baseline image by applying an automatically calculated geometric transformation consisting of translation, rotation, and scale. Unchanged pixels were displayed as white, colder pixels as progressively blue and then black, and warmer pixels as progressively red and then yellow (Supplemental Video 1; supplemental materials are available at http://jnm.snmjournals.org).

### Analysis of PET/CT

Regional estimates of the metabolic rate of glucose [MR(gluc)_BAT_] were calculated as previously described (13). In addition, 2 coronal maximum-intensity-projection (MIP) images for each volunteer were calculated from the PET/CT data for each visit, one for the CT image (CT_MIP_) and one for the MR(gluc) values (MR(gluc)_MIP_) (19). MIP images are 2-dimensional representations of 3-dimensional data, with the value of each point determined by the maximum intensity of the data along a point-line perpendicular to the projected plane, that is:

$${\text{CT}}_{\text{MIP}}\left(x,y\right)={\text{max}}_{\text{z}}\left(\text{CT}\left(x,y,z\right)\right),\text{and}$$

$$\text{MR}{\left(\text{gluc}\right)}_{\text{MIP}}\left(x,y\right)=\max_{\text{z}}\left(\text{MR}\left(\text{gluc}\right)\left(x,y,z\right)\right).$$

A composite of the 2 MIP images was used in the graphical output (Fig. 2). The region of the greatest 10% of MR(gluc)_MIP_ was identified, and the median of these values was calculated for comparison with the results of IRT.

### PET/CT/IRT Comparison

To identify similarities and differences between the data acquired from PET/CT and the data acquired from IRT, the TI was warped onto the MR(gluc)_MIP_ image from the same participant (Fig. 2). The comparative TI was a mean composite of the last 3 images from the initial imaging session (i.e., after 10 min of stimulation) (Figs. 2A and 2B). The TI and MR(gluc)_MIP_ images were displayed beside each other, and control-point pairs were defined, identifying corresponding anatomic points. Seventy control-point pairs were identified in the supraclavicular region of each pair of images, with additional nonsupraclavicular points depending on the framing of the images. A locally weighted mean transformation (20) mapping was created by inferring a second-degree polynomial at each control-point based on the 16 closest points and using a locally weighted average of these polynomials. This mapping was applied to the TI (Fig. 2C).

An ROI was identified on the MR(gluc)_MIP_ defined by applying the locally weighted mean transformation to the contour of the ROI of the TI and superimposing this on MR(gluc)_MIP_. The greatest 10% of values within the ROI were identified for the MR(gluc)_MIP_, and the median of these points was calculated in a similar manner to that described for the TI (Fig. 2D).

The percentage spatial overlap between TI and MR(gluc)_MIP_ hotspots was calculated and displayed as an image (Fig. 2E). Since the TI and MR(gluc)_MIP_ hotspots are, by definition, the same size, the overlap is the number of pixels that the hotspots have in common divided by the total number of pixels of either hotspot).

### Statistical Analysis

After conversion of the TI to the Portable Network Graphic format, further graphical analysis was performed using MATLAB, version 2016a (The Mathworks Inc.). The apices were identified using a custom-built graphic-user interface within MATLAB, and a second script was written to undertake the analysis. The inbuilt MATLAB function *imregister* was used to register sequential images to compile the videos, *cpselect* was used to identify control-point pairs, and *fitgeotrans* was used to fit an lwm transformation object to the control-point pairs. Trends in T_SCR_ over time and correlations between variables were analyzed using R: A Language and Environment for Statistical Computing, version 3.2.3 (R Core Team).

## RESULTS

### TI Analyses

Analyses of TIs from the initial imaging period of the cooling session showed that participants had a base T_SCR_ of 35.1°C ± 0.3°C at the start of stimulation, with a peak temperature of 35.2°C ± 0.3°C during the first 10 min of stimulation (change during first 10 min of stimulation [Δ_10_T_SCR_], 0.1°C ± 0.03°C) (Table 1). Base T_SCR_ was 1.9°C ± 0.2°C above the sternal reference region, with a peak difference of 2.2°C ± 0.3°C during the first 10 min of stimulation (Δ_10_T_SCR_, 0.3°C ± 0.05°C). The peak T_SCR_ of the initial imaging period alone was significantly lower than the peak T_SCR_ during the initial and final imaging periods combined (initial only, 35.2°C ± 0.3°C; both, 35.4°C ± 0.2°C; *P* = 0.03). Similarly, the mean T_SCR_ over the last minute of the initial period of stimulation was significantly lower than the mean T_SCR_ over the last minute of the final imaging session (35.1 ± 0.3°C vs. 35.3 ± 0.2°C, respectively; *P* = 0.03).

**TABLE 1:** IRT Analysis: Absolute T_SCR_ and T_SCR_ Relative to Sternal Reference Region

T_SCR_ (°C)	Vehicle session	Cold session	*P*
Absolute			
Base	35.7 ± 0.07	35.1 ± 0.29	0.09
Peak (initial)	35.8 ± 0.06	35.2 ± 0.29	0.09
Change over first 10 min of cooling	0.1 ± 0.03	0.1 ± 0.03	0.64
Peak (initial and final)	35.9 ± 0.08	35.4 ± 0.25	0.06
At 10 min of cooling	35.6 ± 0.07	35.1 ± 0.30	0.11
In final minute of cooling	35.7 ± 0.08	35.3 ± 0.25	0.09
Relative			
Base	1.3 ± 0.13	1.9 ± 0.24	0.01
Peak (initial)	1.4 ± 0.13	2.2 ± 0.27	0.01
Change over first 10 min of cooling	0.2 ± 0.03	0.3 ± 0.05	0.04
Peak (initial and final)	1.5 ± 0.14	2.7 ± 0.29	0.001
At 10 min of cooling	1.4 ± 0.14	2.2 ± 0.26	0.004
In final minute of cooling	1.4 ± 0.13	2.6 ± 0.27	0.0004

Data are mean ± SEM from 8 men.

There were trends toward a decreased base T_SCR_ from the vehicle compared with the cooling session (vehicle, 35.7°C ± 0.07°C; cooling, 35.1°C ± 0.3°C; *P* = 0.09) and lower peak T_SCR_ (vehicle, 35.8°C ± 0.06°C; cooling, 35.2°C ± 0.3°C; *P* = 0.09) but no difference in Δ_10_T_SCR_ (vehicle, 0.1°C ± 0.03°C; cooling, 0.1°C ± 0.03°C; *P* = 0.64). When compared with the reference region, however, base T_SCR_ (vehicle, 1.4°C ± 0.15°C; cooling, 2.1°C ± 0.23°C; *P* = 0.008), peak T_SCR_ (vehicle, 1.5°C ± 0.15°C; cooling, 2.4°C ± 0.26°C; *P* = 0.006), and Δ_10_T_SCR_ (vehicle, 0.1°C ± 0.03°C; cooling, 0.3°C ± 0.04°C; *P* = 0.007) were all significantly increased by cooling.

### Overlap

Representative images of the process are shown in Figure 2. TI hotspots and MR(gluc)_MIP_ hotspots were in the same anatomic area of the ROIs (Fig. 2E). The overlap between the area of maximal glucose uptake on MR(gluc)_MIP_ and the warmest pixels on TI was 29.5%, respectively (range, 11.6%–55.5%; Table 2). Images from participants who underwent a PET/CT vehicle session demonstrated that, in the absence of cooling activation of BAT, the overlap between the hotspots was significantly less (cooling, 27.9% ± 6.2% overlap; vehicle, 6.3% ± 3.1% overlap; *P* = 0.009) (Table 2).

**TABLE 2:** Percentage Spatial Overlap Between MIP and TI

	Overlap (%)	
Participant	Cold	Vehicle
A	31.6	
B*	11.6	0.1
C*	36.6	8.4
D	55.5	
E	14.3	
F*	38.5	14.1
G*	24.9	2.6
H	22.9	
Mean ± SEM	29.5 ± 5.1	
*Mean ± SEM	27.9 ± 6.2	6.3 ± 3.1

Individual values from 8 men showing percentage spatial overlap between area of maximum supraclavicular MR(gluc) on MR(gluc)_MIP_ image and area of maximum T_SCR_ on TI. Overlap for participants who underwent both cold and vehicle PET/CT scans (*) was significantly reduced in vehicle sessions (cold, 35.4% ± 5.1%; vehicle, 4.7% ± 2.8%, *P* < 0.001).

### Relationship Between Hotspots on PET/CT and IRT

The linearity of the relationship between the IRT and PET/CT outcomes from cooling sessions is given in Table 3. There were strong positive correlations between relative Δ_10_T_SCR_ and both MR(gluc)_MIP_ (*r*^2^ = 0.721; *P* = 0.008) and MR(gluc)_BAT_ (*r*^2^ = 0.583; *P* = 0.027). Absolute T_SCR_ measurements and relative base and peak T_SCR_ did not significantly correlate with MR(gluc)_MIP_ or MR(gluc)_BAT_.

**TABLE 3:** Correlation Between IRT Outcome Variables and PET/CT Measures

Parameter	IRT outcome (right ROI)	MR(gluc)_MIP_	MR(gluc)_BAT_
Relative	Base T_SCR_	0.280 (NS)	0.003 (NS)
	Peak T_SCR_ (initial)	0.386 (NS)	0.032 (NS)
	Δ_10_T_SCR_	0.721*	0.583^†^
Absolute	Base T_SCR_	0.118 (NS)	0.443^‡^
	Peak T_SCR_ (initial)	0.087 (NS)	0.406^‡^
	Δ_10_T_SCR_	0.214 (NS)	0.056 (NS)

* *P* < 0.01.

† *P* < 0.05.

‡ *P* < 0.1.

NS = not statistically significant.

Values are *r*^2^, square of Pearson coefficient for 8 male volunteers.

**FIGURE 3.**
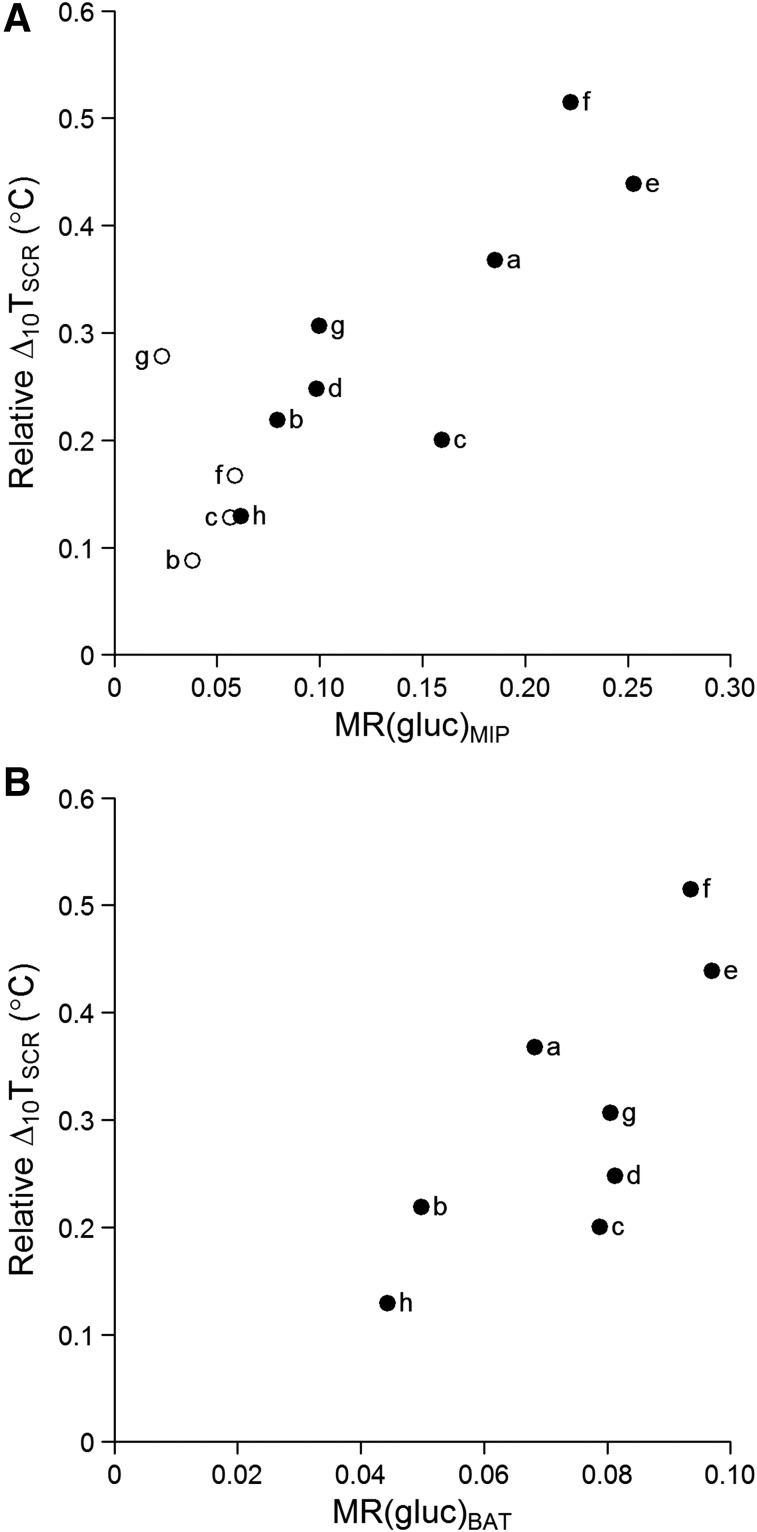
Δ_10_T_SCR_ (relative to sternal reference) against glucose uptake on PET/CT_._ Correlation is shown between BAT activity measured with IRT, median MR(gluc)_MIP_ hotspot value (*r*^2^ = 0.721; *P* = 0.008) (A), and MR(gluc)_BAT_ (*r*^2^ = 0.583; *P* = 0.027) (B). ○ = control; ● = cooling.

All 4 participants who had both cold-activated and vehicle PET/CT scans had an increase in both relative Δ_10_T_SCR_ and MR(gluc)_MIP_ during cooling, compared with the control session (Fig. 3).

Stronger correlations with PET/CT outcomes were seen for the right ROI than the left (Table 4). The left ROI demonstrated a strong positive correlation between MR(gluc)_MIP_ and relative Δ_10_T_SCR_ (*r*^2^ = 0.698; *P* = 0.010) but not with other relative IRT outcomes or with MR(gluc)_BAT_.

**TABLE 4:** Correlation Between IRT Outcome Measures for Left and Right ROIs and PET/CT Measures

PET/CT outcome	IRT outcome (relative to reference region)	Left ROI	Right ROI
MR(gluc)_MIP_	Base T_SCR_	0.172 (NS)	0.280 (NS)
	Peak T_SCR_ (initial)	0.239 (NS)	0.386*
	Δ_10_T_SCR_	0.698^†^	0.721^†^
MR(gluc)_BAT_	Base T_SCR_	0.001 (NS)	0.003 (NS)
	Peak T_SCR_ (initial)	0.002 (NS)	0.032 (NS)
	Δ_10_T_SCR_	0.337 (NS)	0.583*

* *P* < 0.05.

† *P* < 0.01.

NS = not statistically significant.

Values are *r*^2^, square of Pearson coefficient for 8 male volunteers.

## DISCUSSION

IRT is increasingly being used to assess BAT activation and has found an important role in the imaging of healthy volunteers and children, in repeated imaging, and in studies not conducted on fasting subjects (10)—situations in which exposure to ionizing radiation must be minimized. Despite this increasing use, definitive evidence that IRT can measure BAT activity has been lacking. Even studies that have utilized both techniques have done so in a way that does not allow direct comparison (21). We demonstrate here that there is anatomic overlap of the area of maximum temperature measured using IRT and the projected area of maximum glucose uptake on PET. IRT correlates strongly with MR(gluc) from PET/CT. Indeed, since ^18^F-FDG PET/CT measures glucose uptake and not thermogenic capacity per se (22), greater correlations would not reasonably be expected.

The measure of BAT function obtained by PET/CT is therefore different from that obtained by IRT: glucose uptake as opposed to heat production. The question of which measure is the optimum index of BAT function remains to be fully answered. We observed that subjects who were BAT-positive on PET/CT exhibited a similar magnitude of BAT function on IRT. Furthermore, PET/CT is usually conducted on fasting subjects, because after the subject eats, uptake in BAT is largely masked by the much greater uptake in skeletal muscle (10). It has been suggested that eating, per se, stimulates BAT function (23), and to date, we have been able to detect a positive IRT result in all subjects measured (*n* > 200). Taken together, these findings indirectly support those from repeated PET/CT, in which most subjects appear to be BAT-positive on at least one scan (24). In addition, as with the reduced BAT activity in obese adults as measured with PET/CT (25) we have previously observed a reduction in BAT activity with increasing BMI percentile in children (11). Future work comparing differences between IRT and PET/CT measures may indeed offer novel insights into different components of BAT activation.

Previous papers have often used absolute T_SCR_ as the primary outcome. However, many factors may affect T_SCR_ and even cause it to fall (18). In many cases, the magnitude of the effect on T_SCR_ is sufficient to overcome any counteracting factors, but it is clear that true, relative warming is best demonstrated by comparing the T_SCR_ with a reference region. We have used a region on the sternum, but alternatives, such as mean skin temperature (18), may allow more subtle effects to be revealed. However, even with the addition of a simple reference region, the true relationship between the two measures of BAT activity becomes evident. This is perhaps not surprising when videos of change in skin temperature relative to baseline are viewed (Supplemental Video 1). These show the specific warming of the supraclavicular region relative to surrounding skin temperatures and, therefore, the importance of framing the participant to include the superior portion of the sternum as a reference.

We have previously seen a greater rise in the T_SCR_ of the right ROI (11). In addition, we show here that the outcome measures using the right ROI correlate better with BAT glucose uptake on PET/CT than do the outcome measures using the left ROI or a combined measure. The reasons merit further investigation and could be due to either anatomic or functional differences. There is no evidence of a difference in the volume of BAT between the left and right sides on PET/CT (26), but functional symmetry has not been investigated in non-IRT modalities, which could reveal whether asymmetry represents a true difference in activity or, for instance, an artifact or a slight difference in the anatomic position of BAT between the left and right sides.

Base T_SCR_ was measured as an average of the first minute of stimulation. That T_SCR_ is already higher in participants during the cold session demonstrates the rapidity of the BAT response to a cold stimulus. We show here that the response seen within the first 10 min is sufficient to ascertain an individual’s BAT activity, correlating well with glucose uptake on PET/CT; however, contrary to previous reports (12), prolonged stimulation is associated with a further rise in T_SCR_. In addition, a proper acclimatization period is essential for the accurate measurement of BAT activity using IRT and our data (not published) show that this period should be 20 min, during which time the participant should be in the same environment and clothing as for the study and remain as still as during imaging.

The area of maximal glucose uptake on a coronal projection MIP image from ^18^F-FDG PET/CT closely corresponds to the warmest area within the supraclavicular ROI measured using IRT. In the 8 participants known to be BAT-positive on PET/CT, a high degree of overlap was demonstrated between the most intense glucose uptake on PET/CT and the warmest area on IRT.

The main limitation of these analyses is the small number of participants available, reducing the power to detect correlations between IRT and PET/CT outcomes. Larger studies are not ethically feasible, especially in healthy volunteers, because of the risks of exposure to the ionizing radiation associated with PET/CT. We recommend that studies using IRT to measure BAT activity should report the right-sided ROI relative to a reference region, and authors should include a statement about the acclimatization period that was used.

Advances in IRT are allowing for more detailed assessments of changes in superficial temperature associated with changes in the underlying BAT. Radiometric sensors are becoming increasingly accurate, with some sensors now able to distinguish a change of only 0.01°C, and the resolution of images is improving, with cameras able to acquire truly high-definition images. These improvements will allow the subtler changes to be better defined, especially when combined with more sophisticated calculations of reference values for mean skin temperature. To take full advantage of these improvements, together with improvements in image acquisition rates, a fully automated analysis method should be considered. In addition, an automated standardized analysis method would reduce variability between groups. The establishment of IRT as a valid method of measuring supraclavicular BAT activity in humans will make studies feasible that until now have not been possible.

## CONCLUSION

IRT provides a safe, credible, and quantifiable alternative to PET/CT that can now be used in a wide range of population groups for the measurement of BAT activity.

## DISCLOSURE

No potential conflict of interest relevant to this article was reported.
